# Editorial: Pharmacological and Biochemical Perspectives of Kinase Inhibitors in Cancer and COVID-19 Therapeutics, Volume I

**DOI:** 10.3389/fphar.2022.916324

**Published:** 2022-06-15

**Authors:** Balakumar Chandrasekaran, Muthupandian Saravanan

**Affiliations:** ^1^ Department of Pharmaceutical Chemistry, School of Pharmacy, ITM-University, Gwalior, India; ^2^ AMR and Nanotherapeutics Lab, Department of Pharmacology, Saveetha Dental College, Saveetha Institute of Medical and Technical Sciences (SIMATS), Chennai, India

**Keywords:** COVID-19, kinase inhibitors, CDK, ligand-based and structure-based drug design, pharmacology

Kinases are the component of superfamily enzymes known as phosphotransferases which involve transferring a phosphate group from phosphate-releasing high energy molecules to the specific substrates (Ardito et al., 2017). Kinases have significant roles in cell signalling, metabolism, cell transport, protein regulation, and other cellular events (Cohen, 2001). Kinases are the most essential drug-targets participating in 30% of drug design and discovery programs of pharmaceutical industries and are majorly employed as anticancer agents (Bhullar et al., 2018). In general, kinase inhibitors ameliorate life-threatening symptoms of COVID-19 such as cytokine suppression, anti-inflammatory, and antifibrotic activity (Weisberg et al., 2020; García-Cárceles et al., 2022). Several kinase inhibitors reported in the literature were investigated as potential agents for the treatment of COVID-19 complications by targeting enzymes like ABL, SRC, NAK, EGFR, CDK, MAPK, and AKT (Cherukupalli et al., 2018, 2019; McIntosh et al., 2021). Small molecule inhibitors of casein kinase two prevent viral replication since SARS-CoV-2 hijacks this host cell enzyme to achieve infectivity and replication (Borgo et al., 2021). Specifically, JAK2 inhibitor fedratinib was examined for its capacity to suppress TH17-related cytokine storm and proved to be relevant for the treatment of COVID-19 (Melikhov et al., 2021). The drug imatinib, a tyrosine kinase inhibitor, is currently under investigation for COVID-19 treatment as a single agent. However, the combination of imatinib with specific antiviral agents evidenced a synergistic therapeutic outcome in clinical trials involving COVID-19 patients (Ng et al., 2021). Several kinase inhibitors bind to virus-associated proteins that were implicated in developing COVID-19 symptoms such as inflammation, fibrosis, and pneumonia (Defnet et al., 2020; Satarker et al., 2021; Solimani et al., 2021).

The novel Coronavirus Disease 2019 or COVID-19 is caused by Severe Acute Respiratory Syndrome Coronavirus 2 (abbreviated as SARS-CoV-2) has become a universal outbreak and primary life-threatening disease at this point in time (Mahmud et al., 2021; Sankar et al., 2021; Vivekanandhan et al., 2021). For the management of COVID-19, some of the vaccines were developed and quite a few of them are under current development. Pharma and biotech companies are working to identify antiviral drugs against COVID-19, some of which are already indicated as effective agents against other diseases (Bezbaruah et al., 2021; Borah et al., 2021b, 2021a). The clinically valuable vaccines were developed by companies such as Moderna/National Institutes of Health, Pfizer/BioNTech/Fosun Pharma, Johnson and Johnson, AstraZeneca/University of Oxford, and Novavax (Gavriatopoulou et al., 2021). On the other hand, new antiviral drugs were also discovered to manage COVID-19, such as Nirmatrelvir 300 mg with Ritonavir 100 mg, Remdesivir 200 mg, and Molnupiravir 800 mg.

Further, monoclonal antibodies were developed to trigger the immune system to attack a specific invader, SARS-CoV-2. Monoclonal antibodies like Sotrovimab and Bebtelovimab were significant agents for treating COVID-19. This research topic aims to compile original research articles and reviews related to in silico design and synthesis of novel kinase inhibitors applicable in COVID-19 therapeutics by effective modulation of immune responses. About seven potential articles were published on the topic, ‘pharmacological and biochemical perspectives of kinase inhibitors in cancer and COVID-19 therapeutics.

Mahanta et al., employed a computational approach to identify a novel molecule against SARS-CoV-2. This study focuses on the immunological perspective of the infection, involving the SARS-CoV-2 Membrane Glycoprotein (M protein), which focuses on the popularly studied receptor targets. In this study, 7,832 chemical structures from 32 medicinal plants were considered for screening against the homology model of the structure of SARS-CoV-2-M protein. This in silico study identified the compound ZIN1722 as a potential inhibitor to the SARS-CoV-2 M protein, which may subsequently prevent the immunosuppression in the human body during the COVID-19. Naik et al., highlighted the potential roles of kinase inhibitors in reducing COVID-19 symptoms and enhancing patient healthcare systems.

Furthermore, the authors incorporated sufficient information on FDA-approved kinase inhibitors studied through various clinical trials underway, completed, or terminated. Inhibitors including imatinib, ruxolitinib, silmitasertib, and tofacitinib (alone and in conjunction with hydroxychloroquine), are now in clinical studies to determine their efficacy as possible anti-COVID drugs. Ghosh et al., identified potential tyrosine kinase inhibitors by virtual screening of natural compounds from the olive through the OliveNet^TM^ database containing inhibitors of ALK and BTK kinases. Virtual screening was carried out using the ligand-based QSAR and the structure-based docking approaches. The virtually screened library of 161 natural compounds from olive was found to be overexpressed in ALK and BTK in metastatic and virus-host cells. Vimalraj et al., aimed to analyze the expression of long noncoding RNA (lncRNA) metastasis-associated lung adenocarcinoma transcript 1 (MALAT1) in human osteosarcoma (OS) cells and investigated its role in OS-induced angiogenesis. The functional analysis indicated that MALAT1 enhanced OS-induced angiogenesis, *in vitro* and *in vivo* analyses, endothelial cell proliferation and migration, chick embryo angiogenesis assay, and zebrafish xenograft model. The authors suggested that MALAT1 promotes angiogenesis by regulating the miR-150-5p/VEGFA signalling in the OS microenvironment. Khayat et al., studied tirbanibulin, a first-in-class dual Src kinase (non-ATP competitive)/tubulin inhibitor as there are no literature reports available for its structure-activity relationships (SARs). The study is based on the replacement of the outer ring of the biphenyl system resulting in the identification of the pharmacophore of KX chemotype, with a heterocyclic ring. The newly synthesized compounds showed a range of activities in cell-based anticancer assays, which yielded a clear SAR profile. The most potent compound demonstrated cytotoxic IC_50_ values at 294 and 362 nM against HCT116 colon cancer and HL60 leukaemia cell lines, respectively. The ADME profiling of this compound confirmed its good drug-like properties. Gautam et al., evaluated diethylnitrosamine (DEN)-induced HCC in male albino Wistar rats was treated with Dimethyl itaconate (DI) as an anticancer drug. The function and molecular mechanism of DI against HCC *in vivo* were assessed using histopathology, enzyme-linked immunosorbent assay, and western blot studies. Serum metabolomics investigations using ^1^H-NMR revealed that aberrant metabolites in DEN-induced HCC rats were restored to regular following DI therapy.

Furthermore, the data revealed that the DI worked as an anti-HCC agent and was equivalent to that of the commercial chemotherapeutic drug 5-fluorouracil. Singla et al., studied the involvement of kinases and kinase inhibitors in the pathogenesis and treatment of endometrial cancer. The authors discussed the current literature describing natural products derived kinase inhibitors in managing endometrial cancer.
